# Assessing the Performance of RGB-D Sensors for 3D Fruit Crop Canopy Characterization under Different Operating and Lighting Conditions

**DOI:** 10.3390/s20247072

**Published:** 2020-12-10

**Authors:** Jordi Gené-Mola, Jordi Llorens, Joan R. Rosell-Polo, Eduard Gregorio, Jaume Arnó, Francesc Solanelles, José A. Martínez-Casasnovas, Alexandre Escolà

**Affiliations:** 1 Research Group in AgroICT & Precision Agriculture, Department of Agricultural and Forest Engineering, Universitat de Lleida (UdL)–Agrotecnio Centre, Lleida, 25198 Catalonia, Spain; jordi.llorens@udl.cat (J.L.); joanramon.rosell@udl.cat (J.R.R.-P.); eduard.gregorio@udl.cat (E.G.); jaume.arno@udl.cat (J.A.); 2 Department of Agriculture, Livestock, Fisheries and Food, Generalitat de Catalunya, Lleida, 25198 Catalunya, Spain; fsolanelles@gencat.cat; 3 Research Group in AgroICT & Precision Agriculture, Department of Environmental and Soil Sciences, Universitat de Lleida (UdL)–Agrotecnio Centre, Lleida, 25198 Catalonia, Spain; joseantonio.martinez@udl.cat

**Keywords:** RGB-D, depth cameras, precision agriculture, plant phenotyping, agricultural robotics, Kinect v2, time-of-flight sensors, 3D sensors

## Abstract

The use of 3D sensors combined with appropriate data processing and analysis has provided tools to optimise agricultural management through the application of precision agriculture. The recent development of low-cost RGB-Depth cameras has presented an opportunity to introduce 3D sensors into the agricultural community. However, due to the sensitivity of these sensors to highly illuminated environments, it is necessary to know under which conditions RGB-D sensors are capable of operating. This work presents a methodology to evaluate the performance of RGB-D sensors under different lighting and distance conditions, considering both geometrical and spectral (colour and NIR) features. The methodology was applied to evaluate the performance of the Microsoft Kinect v2 sensor in an apple orchard. The results show that sensor resolution and precision decreased significantly under middle to high ambient illuminance (>2000 lx). However, this effect was minimised when measurements were conducted closer to the target. In contrast, illuminance levels below 50 lx affected the quality of colour data and may require the use of artificial lighting. The methodology was useful for characterizing sensor performance throughout the full range of ambient conditions in commercial orchards. Although Kinect v2 was originally developed for indoor conditions, it performed well under a range of outdoor conditions.

## 1. Introduction

The development of sensors capable of acquiring colour and depth data at high frame rates, known as RGB-D sensors, has represented a revolution in the way in which the environment is modelled [1]. While these devices have lower resolutions than either light detection and ranging (LiDAR) sensors [2] or structure-from-motion (SfM) techniques [3], their capacity for 3D imaging without having to move the sensor, combined with their low price, has led to them being deployed with increasing frequency. According to their specific operating principles, RGB-D sensors can be classified into structured-light (SL), time-of-flight (ToF) and active stereoscopic cameras. SL cameras emit infrared laser light that generates a pattern through a diffraction grating that is then projected onto the target, where this light pattern is deformed. Comparisons between the original and the projected pattern allow disparity maps to be generated from which it is possible to compute depth data [4]. ToF cameras are based on the emission of continuous-wave amplitude-modulated light (CW-AM). The distance from the target is determined by the phase shift between the transmitted and reflected signals [5]. Stereoscopic cameras provide 3D reconstruction by combining images (stereo matching) taken simultaneously using binocular or, alternatively, two monocular cameras. Active stereoscopic cameras also include a projector that emits a light pattern, which helps the process of stereo matching in low-textured scenarios [6].

In the field of agricultural research, RGB-D sensors have been used for a number of purposes, including fruit detection [7,8], agricultural robotics [9,10] and weed detection and classification [11,12]. The present work focuses on the use of RGB-D sensors to characterise plant geometry, an activity that seeks to obtain three-dimensional models of crops and to quantify their structural parameters. Initial work carried out in this area has mainly focused on plant phenotyping under indoor conditions using SL cameras. Chéné et al. [13] assessed the potential for using these cameras in leaf segmentation and measuring their curvature and orientation. These sensors have also been used for plant leaf segmentation under greenhouse conditions [14] and to digitize the stems and leaves of different greenhouse tomato plants [15]. Nock et al. [16] highlighted the difficulties involved in detecting small branches using an SL camera. This result was in accordance with [17], where authors concluded that these cameras provide more reliable results when volumetric objects (fruits) are captured. Various studies have also been carried out under field conditions, which have shown the inability of SL cameras to measure plants in daylight [18]. A survey of the potential applications of SL sensors in agriculture and livestock was presented by [19]. The authors concluded that it is necessary to either work under low-light conditions (at sunset or dawn, or on cloudy days) or at night, with the help of artificial lighting. The effect of natural lighting on SL cameras was also discussed in [20], who studied the ability of these sensors to estimate biomass in poplar plantations using different viewing angles, while in [21] the same authors determined the structural parameters of cauliflower crops.

The development of ToF-based RGB-D sensors capable of working outdoors has promoted their use in many 3D crop characterization studies [22]. These included trials in maize (*Zea mays* L.) plants to determine architectural traits, such as plant height and orientation, leaf angle, and stem diameter and position [23,24,25,26]. Accurate 3D models of vineyards have also been generated using a mobile terrestrial laser scanner (MTLS) based on a combination of a ToF camera and a real-time kinematic (RTK) global navigation satellite system (GNSS) [27]. In a close application, known initial and final positions of vineyard rows were used to correct 3D data provided by the ToF camera [28]. In the case of tree plantations, [29] studied associated errors in RGB-D images at different wind speeds, while [30] used depth and pseudocolour images from a ToF camera to supply a convolutional neural network for branch detection in apple trees. New RGB-D sensors based on active stereoscopy have recently been used to estimate canopy volume and bunch detection in a commercial vineyard [31]. These sensors have also been applied to the reconstruction of 3D models of fruit trees using semantic relationships between each side of the tree row [32]. Active stereoscopic cameras are generally more compact and consume less power than ToF cameras.

Previous works have shown that ToF and active stereoscopic cameras are much more robust to variations in lighting than those based on structured light. Even so, and as shown in the comparative study conducted in [33], the performance of all RGB-D sensors is adversely affected by direct sunlight, reducing the number of pixels with valid measurements and, thus, the number of points in 3D point clouds. Therefore, the selection of an RGB-D sensor to be used for 3D orchard characterization requires a good knowledge of the lighting range within which the sensor will operate effectively and efficiently. It should be noted that lighting outside this range could reduce point cloud definition, leading to erroneous estimates of the structural parameters, while reduced lighting would affect the quality of the RGB images.

This work proposes a methodology for characterizing RGB-D sensors in terms of their performance under different lighting conditions and at different distances from the measured target. The methodology presented can be applied to any RGB-D sensor, although a Microsoft Kinect v2 (Microsoft Corporation, Redmond, WA, USA) sensor was used for validation. This is a very popular ToF camera whose introduction has had a revolutionary impact on robotics and industrial uses due to the low cost of obtaining radiometric and geometric data. The novel contributions of the present work with respect to other methodologies listed in the bibliography are the wide range of assessed lighting conditions; the use of new metrics and evaluation parameters, such as those used to evaluate spectral features (RGB colour and NIR intensities), and the provision of the processing code and the acquired dataset to facilitate the use of the present methodology in future works. The rest of the paper is structured as follows: Section 2 presents the sensor used and explains the experimental layout and the parameters used for sensor evaluation; Section 3 evaluates the Kinect v2 sensor by applying the proposed methodology; Section 4 discusses the results and compares them with those obtained in other state of the art research and, finally, the main conclusions are detailed in Section 5.

## 2. Materials and Methods

### 2.1. Experimental Set-Up

The RGB-D sensor evaluated was the Microsoft Kinect v2. This sensor is composed of an RGB camera registered with a near infrared (NIR) ToF sensor to provide RGB, depth and NIR data according to the specifications shown in Table 1.

Data acquisition was performed using ad hoc software developed in the C# language based on Kinect for Windows SDK v2.0. For each capture, this software saves the corresponding RGB image, NIR image and 3D point cloud. The average time required to acquire a single capture, generate the 3D point cloud and save the data, was 1.5 s, using a 64-bit operating system with 8 GB of RAM and an Intel^®^ Core™ i7-4500U processor (1.80 GHz, boosted to 2.4 GHz). In addition, a DVM 1300 lux meter (Velleman, Gavere, Belgium) was used to measure the lighting conditions of each acquired frame. This measurement was carried out by placing the lux meter in an upwards horizontal position at the centre of the captured scene. The wind speed conditions during the experiment were obtained from the Spanish Meteorological Agency (AEMET) service; data were obtained from the closest weather station, which was located 350 m away from the trial site. According to [29], wind speeds of less than 5 m s^−1^ do not affect measurements taken by RGB-D sensors. Since the wind speed measured during the tests was lower than, or similar to this value, its effect was not considered in the evaluation of the results.

The generated 3D point clouds consisted of up to 217 088 points (512 × 424) with local Cartesian coordinates relative to the sensor origin, RGB data and NIR intensity (digital number, DN). Since RGB and NIR sensors have different fields of view (FOV), the left and right RGB image boundaries were not represented in the 3D point cloud. Similarly, top and bottom points of the cloud lacked RGB data. Those points without RGB data were discarded in the colour assessment sections of the methodology.

**Figure 1:**
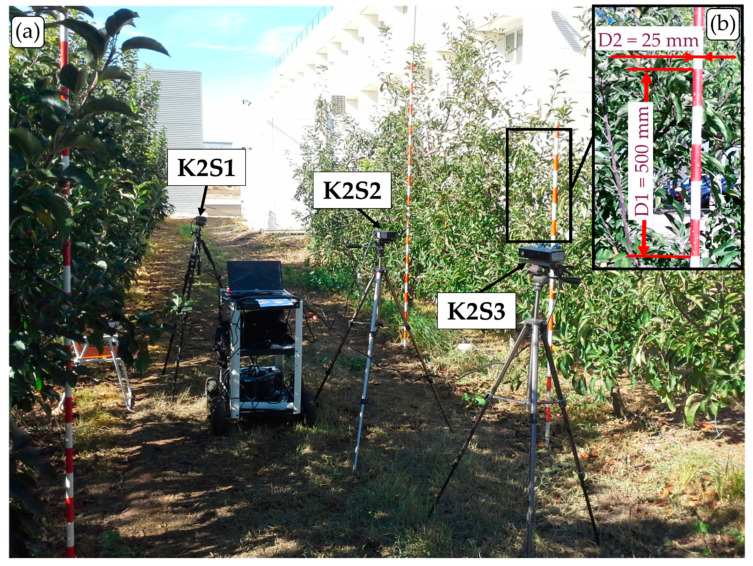
(**a**) Experimental *Malus domestica* Borkh var. Fuji apple orchard at the full vegetation development stage where the measurements were carried out. (**b**) Ranging rod used to evaluate sensor accuracy by means of distances D1 and D2.

All the data used in this work were acquired under real field conditions at the experimental orchards of the School of Agrifood and Forestry Science and Engineering at the Universitat de Lleida. More specifically, measurements were carried out in a plot of apple trees, *Malus domestica* Borkh var. Fuji, at the full vegetation development stage (Figure 1a). The tree canopy was around 3 m tall and 1.5 m wide; the row spacing was 4.5 m, and the tree spacing was 0.75 m. The rows were oriented in an approximately east-west direction. Since sensor performance (resolution, accuracy, precision) mainly depends on the sensor itself and on the scanning conditions, and not on the fruit tree variety measured [19], it is thought that results obtained in an apple orchard should also be applicable to other fruit crops with similar vegetation characteristics.

The proposed methodology includes two different experiments. The first aims to assess the performance of RGB-D sensors under different illumination conditions, while the second seeks to study the performance of the sensor when measuring from different distances to the target. All the data acquired during the experiments, called the Kinect Evaluation in Orchard conditions dataset (KEvOr Dataset), has been made publicly available at https://doi.org/10.5281/zenodo.4286460 [34].

**Figure 2:**
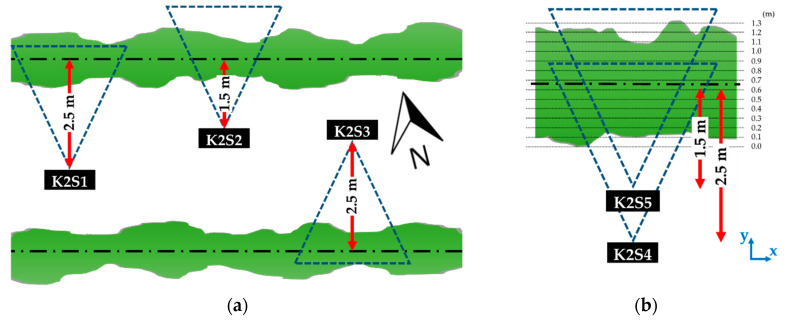
Experiment layout to assess (**a**) the effect of direct (K2S1 and K2S2) and indirect (K2S3) sunlight on the canopy on the generated point clouds; (**b**) the effect of the distance from the target on the generated point clouds. K2Si refers to each of the Kinect v2 (K2) stations. Green areas represent a top view of the tree rows, the dash-dotted line shows the axis of the row, while the thinner dotted lines that have been drawn in (**b**) represent canopy depth intervals (every 0.1 m).

**Figure 3:**
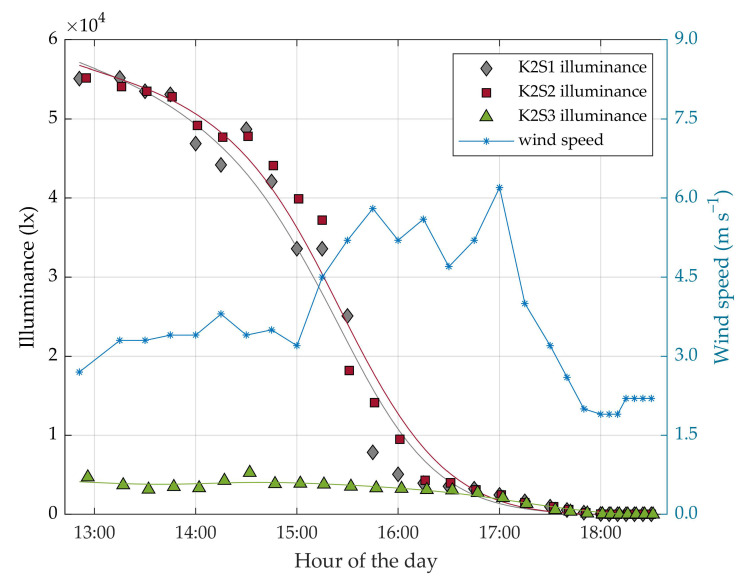
Illuminance (left axis) and wind speed (in blue, right axis) measured throughout the experiment (UTC + 1). Grey diamonds, red squares and green triangles correspond to the illuminance of the measured scene for all K2S1, K2S2 and K2S3 observations, respectively. A regression curve has been added to each illuminance group of values to better compare its trend.

The measurements carried out during the first experiment were taken from three sensor stations (K2S1, K2S2 and K2S3) equipped with three different sensors. The fact that the three sensor stations, which were located at different sites in the orchard, remained static throughout the experiment was important for the acquisition and comparison of the same scenes under the different lighting conditions. K2S1 was oriented approximately to the north and located 2.5 m from the tree row axis, K2S2 was also oriented approximately to the north and located 1.5 m from the tree row axis and K2S3 was oriented approximately to the south and located 2.5 m from the tree row axis (Figure 2a). Thus, K2S1 and K2S2 measured the side of the tree row receiving direct sunlight during the experiment, while K2S3 measured the side of the tree row under indirect sunlight. This distribution allowed the illuminance conditions to be compared, as well as a study of whether the different illuminance levels had the same effect when measuring from different distances. A ranging rod was placed in each measured scene as a reference target for evaluating the accuracy of the sensors (Figure 1b). This experiment started at 12:51 h (November 6, UTC + 1) and consisted of acquiring point clouds at 28 different natural illumination levels throughout the afternoon and evening, from the highest sun illuminance until darkness. Figure 3 shows wind speed and illuminance conditions measured throughout the experiment. For each of these 28 observations, three captures (hereafter replicates) were acquired with each sensor, resulting in a total of 252 captures: 28 lighting conditions × 3 sensors × 3 replicates per sensor. Although two replicates per observation would have been enough to evaluate the sensor precision, three replicates were acquired for added security against a lack of data in the event of a corrupted file. Finally, all replicates were used to evaluate the sensor precision in a more accurate way.

The second experiment was performed on a different day and at a different site within the orchard. A Kinect v2 sensor was stationed 2.5 m from the tree row axis and 1.35 m above the ground (K2S4). A point cloud was acquired and, immediately after, the sensor was placed 1.5 m from the tree row axis to capture the same scene (K2S5) (Figure 2b). This experiment was carried out on 23 July at 20:30 (UTC + 2), when the measured scene received sunlight illuminance of 2000 lx. Due to the different distances to the target, part of the scene measured from K2S4 was out of the K2S5 FOV. To compare the 3D models captured from both stations, the two resulting point clouds were manually registered (aligned) into the same reference systems. The point cloud acquired from station K2S4 was then cropped to match the scene captured from K2S5.

### 2.2. Evaluation Parameters

The present methodology analysed both geometrical and spectral data. Table 2 summarizes the parameters and metrics used in this evaluation. Regarding the geometrical assessment, the point cloud resolution, accuracy, precision and penetrability parameters were studied under different illumination conditions and at different distances from the target. On the other hand, the influence of scanning conditions on the spectral data was assessed by analysing the colour, NIR intensity and range-corrected NIR intensity (NIR_c_) of the acquired point clouds. All these parameters and metrics are described in detail in the following sub-sections.

A MATLAB^®^ (R2020a, Math Works Inc., Natick, MA, USA) code was developed to analyse all the data and provide the sensor evaluation results. This code has been made publicly available at https://github.com/GRAP-UdL-AT/RGBD_sensors_evaluation_in_Orchards [35].

#### 2.2.1. Resolution

Although the NIR camera which was used had a resolution of 512 × 424 pixels, the actual sensor resolution decreased due to the different FOV between RGB and NIR cameras, and the fact that depth sensor performance is affected under high lighting conditions [27]. To evaluate this issue, the total number of points P and the mean density δ were calculated for each acquired point cloud. To compute the average point cloud density δ, the algorithm counts the number of neighbouring points $N_{i}$ contained in a sphere of radius R around each point of the cloud $p_{i}$. The average point cloud density is then estimated by applying the following equation:

(1) $$\delta =\frac{1}{P}\sum_{i=1}^{P}\left(\frac{3N_{i}}{4\pi R^{3}}\right).$$

#### 2.2.2. Accuracy

The ranging rods shown in Figure 1b were used to evaluate sensor accuracy. The longitudinal distance *D*1 = 500 mm and the rod diameter *D*2 = 25 mm were manually measured in all K2S1 and K2S2 point clouds. This measurement was performed by computing the point to point distance of two manually selected points using CloudCompare software (Cloud Compare [GPL software] v2.11 Omnia). Since the accuracy of the measurement depended on the points manually selected, each measurement was repeated four times and averaged to obtain the average distances $D1_{s,j}$ and $D2_{s,j}$ for each point cloud. The four measurements were carried out using the first replicate acquired for each lighting condition. Having these measurements, the average accuracy (Ac) and the root mean square error (RMSE) were computed as follows:

(2) $$Ac_{D,s}=\frac{1}{N}\sum_{j=1}^{N}\left|D_{s,j}-D\right|,$$

(3) $$RMSE_{D,s}=\sqrt{\frac{1}{N}\sum_{j=1}^{N}{\left(D_{s,j}-D\right)}^{2}},$$

where D refers to the measured distance *D*1 or *D*2, s refers to the sensor station 1 or 2, j to the different point clouds acquired throughout the experiment and N to the total number of observations (N = 28).

The data acquired with sensor K2S3 were not used for the accuracy assessment since the distance from the tree row axis was the same as for K2S1 (2.5 m), and the maximum illuminance level received by the K2S3 sensor was 5500 lx.

#### 2.2.3. Repeatability

The assessment of repeatability was performed by comparing the three replicates captured at each lighting condition observation. The comparison consisted of computing absolute point to point distances between each point of a cloud and its nearest neighbour in the corresponding replicated point clouds, hereafter called cloud-to-cloud distance differences. For each sensor (s) and lighting condition observation (L), the precision error (Pr) metric was computed as an average of these cloud-to-cloud distance differences:

(4) $$Pr_{s@L}=\frac{1}{2P^{r1}+P^{r2}}\left(\overset{P^{r1}}{\underset{i=1}{\sum }}d_{1,2,i}+\overset{P^{r1}}{\underset{i=1}{\sum }}d_{1,3,i}+\overset{P^{r2}}{\underset{i=1}{\sum }}d_{2,3,i}\right)$$

where $P^{r1}$ and $P^{r2}$ are the number of points in replicates 1 and 2, respectively, and $d_{k,t,i}$ is the distance between a given point i from replicate k and the cloud obtained with replicate t.

Repeatability was also evaluated in terms of the standard deviation of the cloud-to-cloud distance errors:

(5) $$\sigma_{s@L}=\sqrt{\frac{1}{2P^{r1}+P^{r2}}\left(\overset{P^{r1}}{\underset{i=1}{\sum }}{\left(d_{1,2,i}-Pr\right)}^{2}+\overset{P^{r1}}{\underset{i=1}{\sum }}{\left(d_{1,3,i}-Pr\right)}^{2}+\overset{P^{r2}}{\underset{i=1}{\sum }}{\left(d_{2,3,i}-Pr\right)}^{2}\right)}$$

#### 2.2.4. Penetrability

Using the data from the second experimental set-up (Figure 2b), we assessed the ability of the two point clouds taken from different distances to penetrate the canopy. This was done by computing the number of points in each cloud found in different 0.1 m sections arranged along the *y-axis* (Figure 2b) from the outermost part of the canopy (closer to the sensors) to the most distant part, on the other side of the row. The evaluation of penetrability includes the percentage of points found in each section in relation to the total number of points -hereafter called point distribution in depth, the mean point cloud depth and the standard deviation.

#### 2.2.5. Colour and NIR

The RGB colour data for each captured point cloud was transformed to the HSV (hue, saturation and value) space. This transformation allowed us to assess the influence of ambient light on the captured colour in terms of hue (H) related to chroma, saturation (S) and value (V) related to brightness. The average HSV values for all the points in a given cloud were calculated and compared to daylight illuminance.

For the NIR intensity, a range correction was carried out in order to overcome light attenuation with distance [36]. The corrected NIR intensity (${\mathrm{NIR}}_{c}$) was computed for each point by multiplying the NIR values by the squared distance from the sensor:

(6) $${\mathrm{NIR}}_{c,i}={\mathrm{NIR}}_{i}\left(x_{i}{}^{2}+y_{i}{}^{2}+z_{i}{}^{2}\right).$$

where $\left[x_{i},y_{i},z_{i}\right]$ are the Cartesian coordinates of a point i with respect to the sensor.

We calculated average NIR and ${\mathrm{NIR}}_{c}$ values for all the points in a cloud, and also their distribution, using a normalised histogram. These were then compared to analyse the effect of the illuminance conditions and distance from the target on the returned NIR and ${\mathrm{NIR}}_{c}$ light.

#### 2.2.6. Statistical Analysis

Statistical analysis was performed using the statistical software R (R Development Core Team, 2013) under an RSTUDIO environment (ver. 1.2.3001). The effects of the different Kinect positions and orientations plus five illumination levels (Illumination brakes at: 0, 250, 1000, 4000, 16000 and 64000 lx) were examined using two-way analyses of variance (ANOVA) followed by Tukey’s HSD (honestly significant difference) test for multiple comparisons. Assumptions of ANOVA were checked and, according to the model’s diagnosis, Box-Cox transformation was applied. All the statistical analyses were conducted at a significance level of α = 0.05.

## 3. Results

### 3.1. Experiment I: Effect of Daylight Illuminance

#### 3.1.1. Point Cloud Resolution

**Figure 4:**
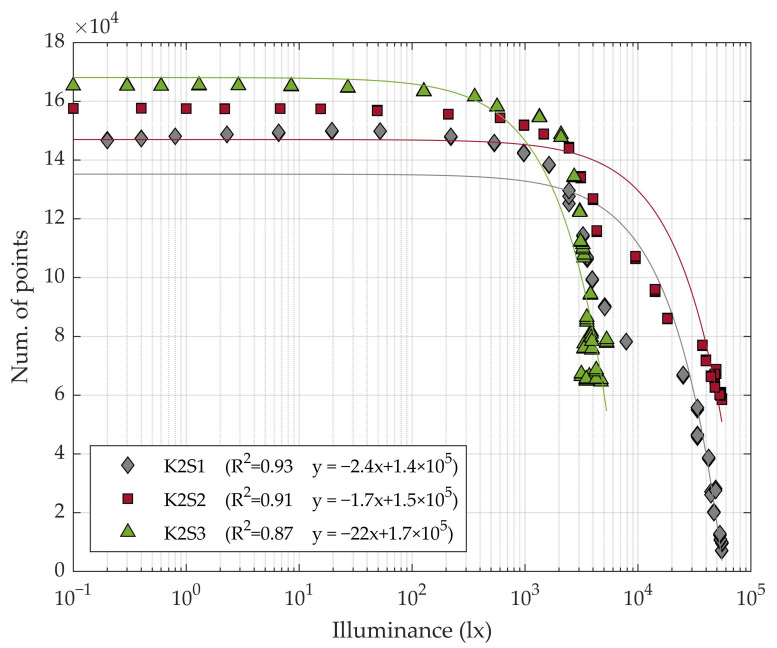
Evolution of the number of points in the point clouds as a function of illuminance.

**Figure 5:**
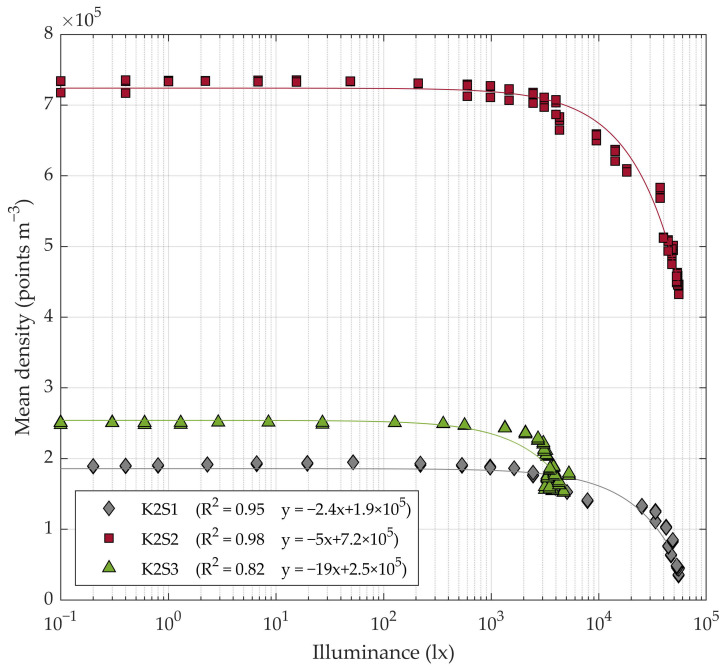
Evolution of average point cloud density as a function of illuminance.

The point cloud resolution remained almost stable for low illuminance levels but started to decrease significantly for middle to high illuminance levels (>2000 lx) (Figure 4 and Figure 5).

The number of points in the cloud showed an inversely linear dependence with the ambient illuminance (Figure 4), reporting coefficients of determination $R^{2}$ of 0.93, 0.91 and 0.87 for K2S1, K2S2 and K2S3, respectively. The sensor station K2S1, which was placed 2.5 m from the tree row axis, showed a higher regression coefficient (or slope *s_i_*) *s*_1_ = −2.4 than K2S2, which was placed at a distance of 1.5 m, which reported a regression coefficient of **s_2_ = −1.7. From this, it was concluded that the illuminance effect was slightly reduced when scanning closer to the target. The K2S3 sensor showed the highest regression coefficient: *s*_3_ = −22. However, this sensor station was the one least affected by the lighting conditions throughout the day. It reported over 60000 points in the cloud even at the time of day when the sunlight was strongest (Figure 4). The higher slope reported with K2S3 may have been due to the fact that the north face of the measured row did not receive the incidence of the sunlight directly. As a consequence, it did not experience large variations in illumination as the position of the sun changed in the course of the day (Figure 3). Even so, backlighting continued to affect sensor performance.

The mean point cloud density also showed an inversely linear dependence on illuminance (Figure 5), reporting $R^{2}$ values of 0.95, 0.98 and 0.82, respectively. This behaviour had been expected, as the FOV of the sensors was constant and the number of points augmented as the light illuminance declined (Figure 4). Another expected result was that the average point cloud density decreased with distance from the target. For instance, under the worst lighting conditions (~50000 lx) K2S2 registered a higher point cloud density $\delta_{2@50klx}>4\times 10^{5}\;{\mathrm{points}\;m}^{-3}$ than K2S1 and K2S3 at low illuminance levels $\delta_{1,3@1lx}<3\times 10^{5}\;{\mathrm{points}\;m}^{-3}$.

**Figure 6:**
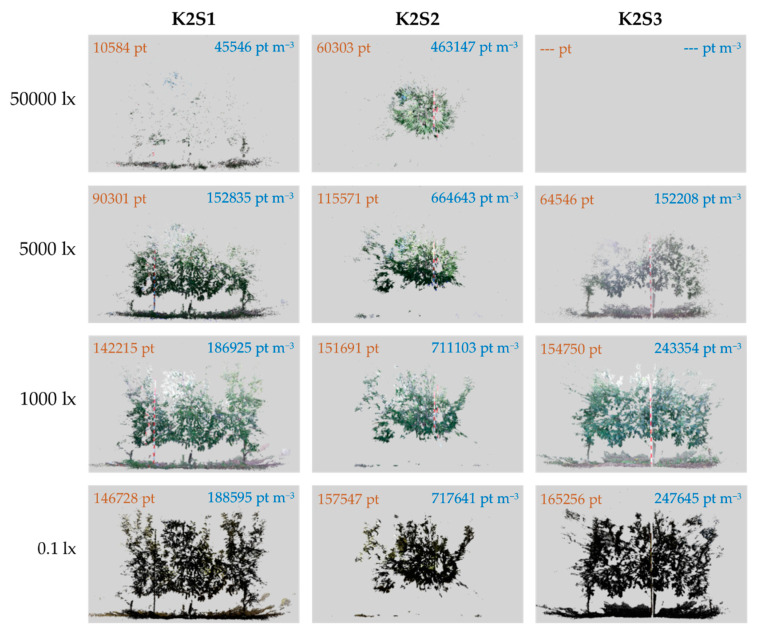
Point clouds acquired from sensor stations K2S1, K2S2 and K2S3 under different illuminance conditions. The orange and blue values correspond to the number of points in the cloud and the average point cloud density for each capture, respectively.

For a qualitative assessment of the illuminance effect, Figure 6 shows examples of the point clouds acquired from each sensor station at different illuminance levels. In addition, the number of points and the point cloud density values are provided for each case (orange and blue values, respectively). Since the K2S3 sensor captured the indirectly illuminated row side, the measurements with this sensor did not achieve an ambient illumination greater than 5500 lx (Figure 3). This explains why no information is given in the K2S3–50000 lx sample.

As can be observed in Figure 6, the K2S1 sensor was strongly influenced by high levels of illuminance, resulting in a sparse 3D model with only a limited ability to represent small objects such as fruits, trunks, branches or leaves. The high illuminance also affected the data captured by the K2S2 station. In this case, the effect was visible in the point cloud boundaries, where the target was further away from the sensor. Thus, although the point cloud density at high illuminance was acceptable for short range measurements (Figure 5), the lighting conditions still affected the performance of the sensor by reducing its FOV. At 5000 lx, the sensor that was most affected was K2S3. This effect was due to the fact that the K2S3 sensor measured the indirectly illuminated side of the row. Although this side did not receive high level of illumination during the hours of strongest sunlight (5000 lx at 13:00 UTC + 1), sensor performance continued to be affected due to the greater amount of backlight. Finally, when comparing the point clouds acquired at 1000 lx and those acquired at 0.1 lx, we observed no significant differences in terms of the geometric evaluation; very similar point clouds were obtained under both lighting conditions.

#### 3.1.2. Point Cloud Accuracy

Sensor accuracy was computed for different lighting conditions, but not all the captures could be analysed. Under conditions of very low illuminance, the lack of colour data prevented the acquisition of reliable measurements because the coloured stripes on the ranging rod could not be differentiated. On the other hand, when the lighting levels were too high, the low point cloud resolution made it impossible to correctly identify the elements at the scene. As a result, the illuminance ranges considered for the accuracy assessment ranged from 2.3 lx to 42100 lx, and from 1 lx to 54100 lx, for K2S1 and K2S2, respectively (Table 3).

After analysing the accuracy results over the specified ranges, we found no relationship between the illuminance of the scene and the accuracy of the sensor and concluded that sensor accuracy was not affected by the lighting conditions. Table 3 reports the mean accuracy and the RMSE obtained for measuring distances D1 and D2. K2S1 showed slightly better performance measuring longer distances (D1 = 500 mm), while K2S2 was slightly more accurate when measuring shorter distances (D2 = 25 mm), but no significant differences were observed. Based on the small differences between the two sensor stations (K2S1 and K2S2), we concluded that there was no clear relationship between sensor accuracy and distances between sensors and row axis of 1.5 m and 2.5 m.

#### 3.1.3. Repeatability of the Measurements

**Figure 7:**
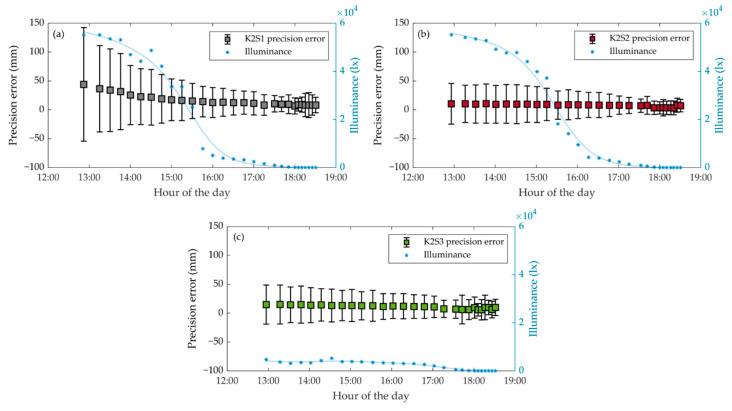
Evolution of point cloud precision as a function of the illuminance conditions throughout the experiment (UTC + 1). The centres of the grey, red and green squares, respectively, indicate the precision errors, Pr, at K2S1 (**a**), K2S2 (**b**) and K2S3 (**c**), while the whiskers correspond to the ± standard deviation.

Repeatability results also showed a dependency on lighting conditions (Figure 7 and Table 4). The most affected sensor station was K2S1, which showed an increase in the precision error with brighter lighting conditions from $Pr_{1@1lx}$ = 8.2 mm to $Pr_{1@55klx}\;$= 44 mm. This station also experienced the highest increase in distance error standard deviation: from $\sigma_{1@1lx}=$ 21.5 mm to $\sigma_{1@55klx}\;$= 98.4 mm. These differences could also be seen in the mean precision error and mean standard deviation in each illumination class (Table 4). Sensor station K2S2 presented the best precision values across the illumination range, and also the least variation in these results.

Within the ranges 0 lx to 250 lx (Class 1) and 1000 to 16000 lx (Class 3 and 4), the precision of the K2S3 results was statistically the same as that for K2S1 (Table 4). Despite this similar behaviour, as the illuminance values throughout the day were higher for K2S1 than for K2S3, the latter was less affected by the time of day with most sunlight: from 12:51 h to 16 h (UTC + 1) (Figure 7). Finally, the precision of K2S2 was the least affected by the lighting conditions. Measuring the scene from a shorter distance significantly improved the precision of the results under high illuminance conditions. This can be clearly observed by comparing K2S2 and K2S1 (Figure 7): at 55000 lx, $Pr_{2@55klx}\;$= 10.6 mm and $\sigma_{2@55klx}\;$= 35.2 mm. This effect was also observed when comparing stations K2S2 and K2S3, but to a lesser extent.

#### 3.1.4. Spectral results: Color and NIR

**Figure 8:**
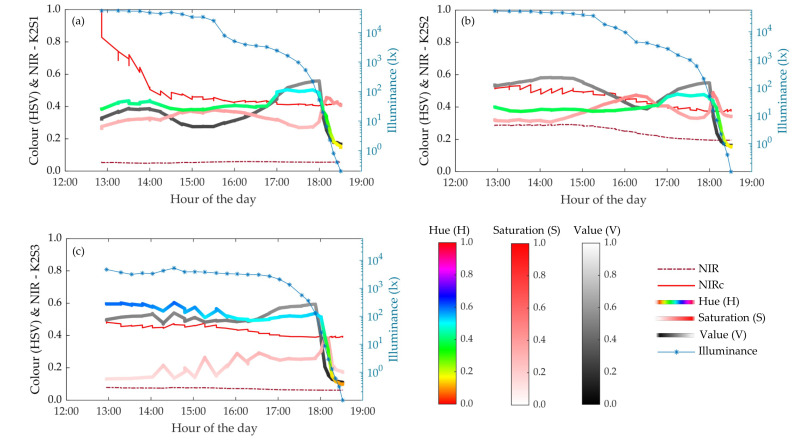
Evolution of the colour indices HSV, NIR and NIRc intensities and illuminance throughout the experiment. The plots of (**a**–**c**) correspond to the results obtained from sensor stations K2S1, K2S2 and K2S3, respectively.

**Figure 9:**
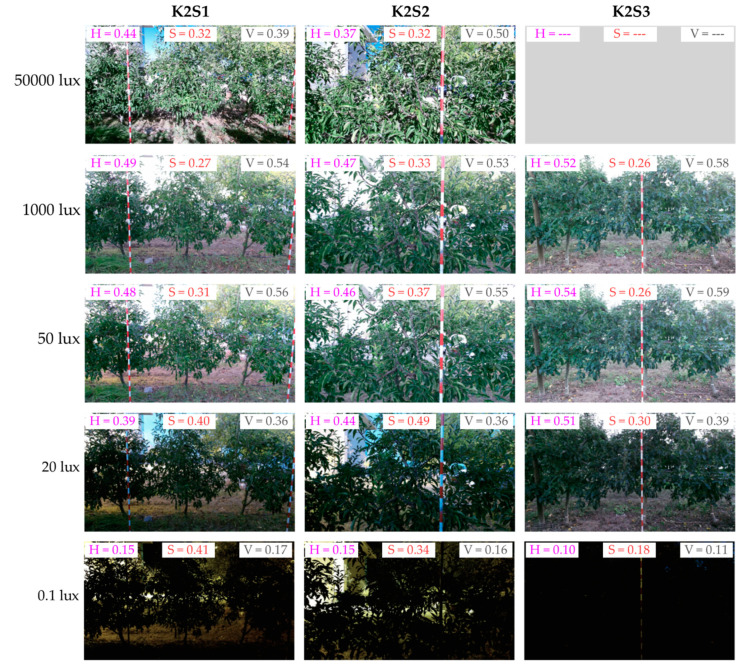
Colour images acquired from the K2S1, K2S2 and K2S3 sensor stations under different illuminance conditions. Pink, orange and grey values correspond to the mean hue (H), saturation (S), and brightness (V) indices of the corresponding point clouds.

The colour camera integrated into the tested RGB-D device showed a good level of adaptation to different lighting conditions above 50 lx. Between 16:30 h and 18:00 h, the mean image brightness (V) increased at all three sensor stations (Figure 8). This behaviour contrasted with the natural lighting conditions, which decreased from 3000 lx to 50 lx (approximately) during this period. Automatic adjustments of the colour sensor explain the difference between the ambient illuminance and the V value. Since the sensor automatically adjusts the image exposure to maintain a good level of colour brightness in the target area, the background image becomes highly exposed (or even burnt) when it measures a shadowed scene. As a result, the highly exposed background increased the mean V. This effect can be observed by comparing the images at 50000 lx and at 1000 lx, in Figure 9.

For lighting conditions below 50 lx (from 18:00 onwards), the illuminance was too low to be corrected by adjusting the sensor exposure. As a result, V started to decrease significantly. This effect can be clearly seen in Figure 8, but it can also be qualitatively observed by comparing the images in Figure 9 in which the measured scene shows similar V levels at 1000 lx and 50 lx, but they significantly decrease with lower ambient illuminance of 20 lx and 0.1 lx.

Hue (H) showed similar behaviour to the V curve (Figure 8). From this it was concluded that images acquired at illuminance levels of less than 50 lx could not be postprocessed to correct low brightness because the image chroma was also affected under such lighting conditions.

For the sake of simplicity in the visualization of the plots, the mean NIR and ${\mathrm{NIR}}_{c}$ (corrected) values plotted in Figure 8 were scaled within the range of 0 to 1. This was done by dividing the NIR and ${\mathrm{NIR}}_{c}$ values by a factor of 15000, which coincides with the maximum ${\mathrm{NIR}}_{c}$ value reported during the experiment. Looking at Figure 8a (K2S1 sensor), the ${\mathrm{NIR}}_{c,1}$ increased significantly at high illuminance levels, coinciding with the illuminance range where the sensor showed low resolution values (Section 3.1.1). Since ${\mathrm{NIR}}_{c}$ is related to an object’s reflectance [37], it can be concluded that the higher the ambient illuminance, the greater the object’s reflectance needs to be measured by the sensor. This effect was minimized when measuring from shorter distances (1.5 m), as can be observed in Figure 8b (K2S2 sensor).

K2S1 and K2S3 showed similar NIR values: between 0.05 and 0.1 (scaled values). This contrasts with the values reported by K2S2, whose NIR values ranged from 0.2 to 0.25. This difference was due to the inverse-square law, i.e., the attenuation of the intensity of light coming from a source expressed as the inverse of the square of the distance from that source. This effect was remedied with the range correction. As can be observed in Figure 8, although ${\mathrm{NIR}}_{1}$ and ${\mathrm{NIR}}_{3}$ are lower than ${\mathrm{NIR}}_{2}$, after range correction, all three values ${\mathrm{NIR}}_{c,1}$, ${\mathrm{NIR}}_{c,1}$ and ${\mathrm{NIR}}_{c,1}$ are similar (between 0.4 and 0.5).

### 3.2. Experiment II: Effect of the Distance from the Target

The geometrical parameter most affected by the distance from the target was point cloud density, which showed an increase of 200.5 % when measured from a distance of 1.5 m, compared to the results obtained from 2.5 m (Table 5). These results are consistent with those reported in the first experiment (Section 3.1.1, Figure 5), which showed a significant difference between sensor stations K2S1 (2.5 m) and K2S2 (1.5 m) throughout the day.

**Figure 10:**
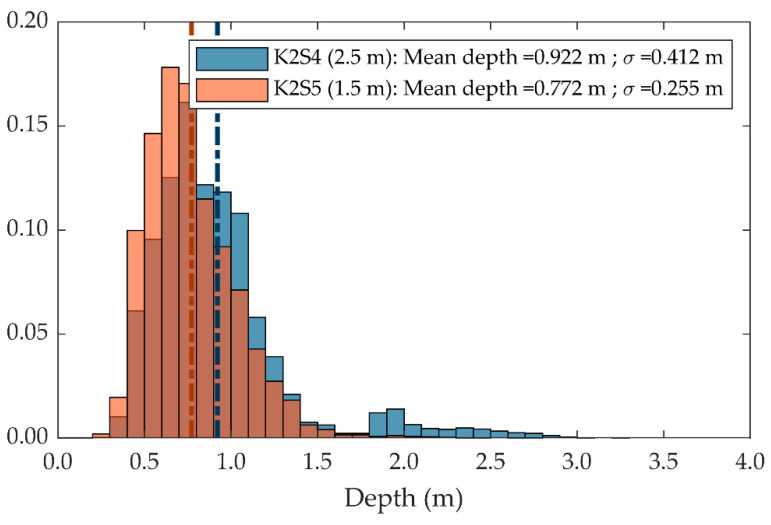
Penetrability of point clouds acquired from 1.5 m (orange) and 2.5 m (blue). The colour brown corresponds to the orange and blue bars overlapping. Vertical dash-dotted lines illustrate the mean depth values, computed as the mean of points’ depth distribution.

While point cloud density decreased with distance, point cloud penetrability improved. As can be observed in Figure 10, points measured from 1.5 m were shallowly distributed compared with those measured from 2.5 m, which were able to penetrate the canopy more deeply. As a result, the mean depth distribution (penetrability) increased from 0.772 m at 1.5 m to 0.922 m at 2.5 m (Table 5). The authors attribute this difference to the fact that the closer the target was to the sensor, the larger were the shadows from the NIR light emitted by the sensor, due to its greater interference with the outer elements of the canopy.

With respect to colour, brightness (V) was the colour parameter that was most affected by distance. It showed an increase of 30.4 % when measured from a distance of 1.5 m compared with results obtained from 2.5 m (Table 5). However, since the hue (H) and saturation (S) values did not present any major differences between 1.5 m and 2.5 m measures, the difference in brightness could be corrected by scaling RGB channels with a scaling factor >1.

**Figure 11:**
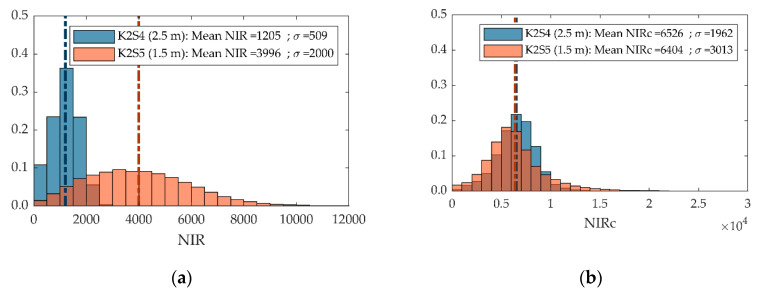
Distribution of the backscattered NIR (**a**) and NIR_c_ (**b**) intensities of point clouds acquired from 1.5 m (**orange**) and 2.5 m (**blue**). The colour brown corresponds to the orange and blue bars overlapping.

As for the NIR intensity values, due to the inverse-square law attenuation of NIR light intensity, the NIR intensity distribution at 1.5 m differed significantly from that at 2.5 m (Figure 11a). The point cloud acquired at 2.5 m showed a mean NIR intensity of 1205 (DN) with a standard deviation of 509 (DN), while at 1.5 m the mean NIR was 3996 (DN) and the standard deviation was 2000 (DN). Since light attenuation depends on the square of the distance [37], the distance effect was overcome after applying range correction (Equation (6)) and obtaining similar mean NIR_c_ values at both of the distances tested (Figure 11b). This same effect was also observed under different lighting conditions in the first experiment (Section 3.1.4, Figure 8).

## 4. Discussion

The first experiment showed that high illumination levels affected the geometrical data acquisition performance while low illumination levels affected the colour data. In the case of geometrical performance, the point cloud resolution and repeatability were the parameters most affected. The number of measured points and the point cloud density both decreased significantly for middle to high illuminance levels (>2000 lx). In contrast, too little illuminance levels affected the quality of the colour data, with performance being poor when lighting levels were below 50 lx. In order to obtain reliable measurements, for both geometrical and spectral data, the lighting level should, therefore, be within the range of 50 to 2000 lx.

The objectives and agronomic applications of RGB-D sensors are highly varied, and high levels of performance are not always required for both geometrical and spectral data. In consequence, different optimal working conditions may be established depending on the application. For instance, if the objective is to find only the geometric characteristics of an orchard [38], or to measure the porosity of its canopy [39], it should be a priority to capture as many points as possible, even if this entails sacrificing the colour data at each point. In such a case, the measurements should be performed in the absence of, or with only low levels, of light. On the other hand, if the main goal is to classify tree elements (such as leaves, fruits, trunks, branches) and/or to assess their condition (e.g., state of fruit ripeness, or water stress) from leaf colour, obtaining sensor conditions that can provide good colour quality while, at the same time, still maintaining a significant number of points should be prioritized. In this case, low illuminance should be avoided.

When both colour and geometric features are important, the hours of the day guaranteeing optimal lighting conditions are very limited. Even so, scanning conditions can be improved by adapting and optimising the experimental layout. One solution could be to plan the captures so that the sensor only measures the indirectly illuminated side of each row when solar illumination is too intense. This was evaluated using sensor station K2S3, which showed acceptable geometrical performance throughout the day, reporting mean point cloud densities that were greater than 1.5 × 10^5^ points m^−3^. Another solution would be to reduce the distance between the sensor and the target. By placing the sensor 1.5 m from the tree row axis (K2S2 and K2S5), the average point cloud density increased, registering mean density values of more than 4 × 10^5^ points m^−3^ throughout the experiment. The main disadvantage of scanning from a shorter distance is that the sensor FOV is reduced significantly. This issue could be solved by using an array of sensors distributed at different heights with a certain degree of overlap and using them to obtain a single point cloud for the whole captured scene [40,41]. Finally, it would be possible to extend the period with adequate lighting conditions by using artificial lighting. This has been considered in many agricultural applications that require high-quality colour data, such as automatic fruit detection [42], weed detection [43] and with harvesting robots [44], among others. Besides extending the period with adequate illumination, the use of artificial lighting optimises the illumination, thereby reducing any undesirable effects such as shadows or specular reflections. In [45], the authors used an over-the-row machine to cover the measured scene, preventing high illuminance from direct sunlight and obtaining better control of the light by means of artificial lighting. This solution could provide optimal lighting conditions throughout the day, but would require a bulky and unwieldy scanning machine.

Although all the tests carried out in this work involved Kinect v2, the method could be applied to evaluate any other RGB-D sensor. Other authors have evaluated the performance of different RGB-D sensors under different measuring conditions. In [46], ten RGB-D sensors were evaluated considering the influence of different target materials, lighting conditions and interference from other sensors, but all of those tests were carried out under indoor lighting conditions at under 535 lx. Similarly to the present work, the Kinect v2 sensor evaluated in [46] did not show any significant differences in accuracy or precision at distances of between 2.5 m and 1.5 m under low illuminance conditions. In [47], three different RGB-D sensors were tested. Outdoor experiments evaluated the sensors from the geometric point of view, considering different materials, shapes, distances, angles of incidence and light intensities ranging from 15000 lx to 100000 lx. Despite there being fewer points in high illuminance environments, the results showed that the Kinect v2 performed well to fit planes and cylinders on geometrical surfaces. These results were consistent with those reported in the present work, as a relatively small number of points tended to be enough to fit a geometrical shape. Despite having a low point cloud resolution, the accuracy of the sensor was not affected by high lighting conditions. Similarly, in [33], four RGB-D sensors were tested to measure young corn plants and tomatoes. The scanning conditions considered included different distances from the target, ranging from 0.2 m to 1.5 m, and four illuminance intensities. The geometrical parameters measured were the fill rate and the RMSE obtained when measuring tomatoes. The RMSE results reported in [33] for Kinect v2 showed an increase with distance, which contrasts with the results obtained in the present work and in [46], in which no significant difference was observed between the accuracy measured at 1.5 m and at 2.5 m. However, these results are not fully comparable since the ranges of distance tested in the two works were different. The precision of the measurements could have been affected by the movement of leaves and branches under high wind conditions. The highest wind speeds were recorded from 15:30 (UTC + 1) to 17:00 (UTC + 1) (Figure 3), with values ranging from 5 to 6 m s^−1^. Even so, no significant differences were observed in the precision error metric during this period. These results were similar to those reported in [29], from which it could be concluded that sensor performance was not affected by wind speeds under 5 m s^−1^.

The proposed methodology includes a comprehensive evaluation of RGB-D sensors and offers certain advantages over methodologies used in previous works [33,46,47]. Firstly, this methodology was specifically designed to evaluate RGB-D sensors in orchard environments. It considers a wide range of lighting conditions, two different sensor orientations and distances from the row axis of 1.5 m and 2.5 m, which is similar to the spacing between the tree row axis and the centre of the alleyway in most commercial orchards. In addition to the geometrical assessment, the present work also includes an evaluation of the colour and NIR intensities, which are important factors in many agriculture applications. Finally, to facilitate the use of the present methodology in future works, with other sensors and under other conditions, the KEvOr dataset and the processing code have been made publicly available at [34] and [35], respectively.

## 5. Conclusions

This work presents a methodology for assessing RGB-D sensors used in fruit tree orchards within the framework of precision agriculture. This was used to evaluate the performance of the Kinect v2 sensor under different scanning conditions. Two different experiments were carried out. The first evaluated sensor performance under different lighting conditions. The results showed that the point cloud resolution remained stable at low illuminance levels, but decreased significantly at middle to high illuminance levels (>2000 lx). This illuminance effect was slightly reduced when measurements were taken closer to the target (canopy), but short-range measurements also reduced sensor FOV, thereby limiting the capacity to characterize trees with a single sensor. Although high illuminance conditions reduced the number of points obtained, the accuracy of the remaining points was not affected. In contrast, since the points obtained at high illuminance differed between replicates, the precision of the measurements was affected at times of high exposure to sunlight. The colour data showed a good adaptation to different lighting conditions above 50 lx, but below this level the illuminance was too low to be corrected by the sensor exposure adjustment.

The second experiment aimed to assess sensor performance at different distances from the target. The results showed that point cloud resolution decreased with distance. This effect was mainly due to the higher FOV of the sensor and the constant number of 3D points measured. In addition, as NIR light intensity decreases quadratically with distance when measuring from long distances, the NIR camera could not distinguish the sensor-emitted light. This attenuation of the light was clearly observed when comparing the NIR intensities obtained from distances of 1.5 m and 2.5 m. To overcome this attenuation of the light, we range-corrected the NIR light and obtained a similar distribution of NIRc intensities when correcting both captures (1.5 m and 2.5 m).

The proposed method was able to evaluate RGB-D sensors in terms of geometrical and spectral features and to consider the different scanning conditions found in commercial orchards. Although the RGB-D sensor that we tested was originally developed to work under indoor conditions, it has been shown that it can also be used under certain specific outdoor conditions. The optimal lighting conditions for using the Kinect v2 in agricultural environments range from 50 lx to 2000 lx. However, this range could be expanded for agricultural applications that do not require full sensor performance. Future works may apply the approach presented here to test other RGB-D sensors and to define their capacity for use in agricultural environments.

## Figures and Tables

**Table 1:** Microsoft Kinect v2 sensor specifications.

RGB Frame Resolution	1920 × 1080 pixels
RGB frame rate	30 Hz
Infrared/Depth frame resolution	512 × 424 pixels
Infrared/Depth frame rate	30 Hz
Infrared/Depth field of view	70° horizontal × 60° vertical
Depth range	0.5–4.5 m

**Table 2:** Evaluation parameters and metrics.

Parameter	Metrics	Units
Resolution	Number of points (P)	points
	Average point cloud density (δ)	points m^−3^
Accuracy	Accuracy (Ac)	mm
	Root mean square error (RMSE)	mm
Repeatability	Precision error (Pr)	mm
	Standard deviation (σ)	mm
Penetrability	Points distribution in depth	%
	Mean point cloud depth	m
	Standard deviation	m
Colour	Mean hue (H)	%
	Mean saturation (S)	%
	Mean brightness (V)	%
NIR	Mean NIR intensity	DN
	Mean NIR_c_ intensity	DN m^2^

**Table 3:** Sensor accuracy results measuring from 2.5 m (K2S1) and from 1.5 m (K2S2) of the tree row axis. In rows, the same letter indicates no significant differences between mean accuracy values (*p* < 0.05).

	K2S1	K2S2
Min. illuminance to measure [lx]	2.3	1
Max. illuminance to measure [lx]	42100	54100
${\mathrm{Accuracy}}_{D1}$ [mm]	3.7 a	6.1 a
${\mathrm{Accuracy}}_{D2}$ [mm]	7.3 a	4.4 a
${\mathrm{RMSE}}_{D1}$ [mm]	6.6	7.5
${\mathrm{RMSE}}_{D2}$ [mm]	9.5	6.0

**Table 4:** Mean values of precision errors and standard deviation at each illumination level with their mean statistical differences. Station K2S3 was not tested at above 16000 lx. In rows, for each parameter, the same letter (Latin letters for precision error and Greek letters for standard deviation) indicates no significant differences between the mean accuracy values (*p* < 0.05).

Class	Lux Range [lx]	Precision Error [mm]			Standard Deviation [mm]		
		K2S1	K2S2	K2S3	K2S1	K2S2	K2S3
1	0 to 250	8.54 a	4.62 b	7.86 a	15.90 α	11.82 β	15.80 α
2	250 to 1000	10.76 a	7.64 b	6.74 c	13.53 β	13.49 β	19.35 α
3	1000 to 4000	11.76 a	7.89 b	12.50 a	20.84 β	16.38 β	24.78 α
4	4000 to 16000	13.43 a	8.61 b	14.14 a	26.17 β	23.28 γ	30.18 α
5	16000 to 64000	25.86 a	9.88 b	--	54.72 α	31.75 β	--

**Table 5:** Geometric and spectral results at different distances from the tree row axis: 2.5 m and 1.5 m.

	K2S4 (2.5 m)	K2S5 (1.5 m)	Difference
Point cloud density [points m^−3^]	215 × 10^3^	646 × 10^3^	200.5 %
Penetrability [m]	0.922	0.772	16.3 %
Hue (H) [%]	46.5	45.9	1.3 %
Saturation (S) [%]	25.5	27.6	8.2 %
Brightness (V) [%]	41.1	53.6	30.4 %
Mean NIR intensity [DN]	1205	3996	231.6 %
Mean NIR_c_ intensity [DN m^2^]	6526	6404	1.9 %
